# Linezolid-induced black hairy tongue: a case report

**DOI:** 10.1186/1752-1947-7-46

**Published:** 2013-02-15

**Authors:** Faisal Abdullah Khasawneh, Dereje Fikremariam Moti, Joseph Anthony Zorek

**Affiliations:** 1 Section of Infectious Diseases, Department of Internal Medicine, School of Medicine, Texas Tech University Health Sciences Center, Amarillo, TX, USA; 2 Department of Internal Medicine, School of Medicine, Texas Tech University Health Sciences Center, Amarillo, TX, USA; 3 Department of Pharmacy Practice, School of Pharmacy, Texas Tech University Health Sciences Center, Amarillo, TX, USA

**Keywords:** Oxazolidinone, Linezolid, Back hairy tongue

## Abstract

**Introduction:**

Linezolid-induced black hairy tongue has been rarely reported. The purpose of this paper is to report a case of linezolid-induced black hairy tongue and review the literature.

**Case presentation:**

A 56-year-old Caucasian man was admitted with community-acquired pneumonia that failed to respond to levofloxacin 750mg daily. He was started on linezolid and meropenem and was subsequently discharged home on oral linezolid 600mg every 12 hours and intravenous ertapenem 1g daily. On a follow-up clinic visit, day 14 of linezolid therapy, he complained of dysgeusia and his tongue examination was consistent with black hairy tongue. After he finished his antibiotic course, his complaints resolved with regular tongue brushing.

**Conclusion:**

Black hairy tongue is characterized by abnormal hypertrophy and elongation of filiform papillae. Five reported cases of linezolid-induced black hairy tongue were identified in a MEDLINE search (from January 2000 to June 2012). The Naranjo Probability Scale revealed a probable adverse drug reaction of linezolid-induced black hairy tongue. Potential contributing factors included other antibiotics, drug–drug interaction and poor oral hygiene. Health care professionals should be aware of the possibility of linezolid-induced black hairy tongue. Thorough history for other possible contributing factors should be obtained. Patients on linezolid should be counseled to perform good oral hygiene.

## Introduction

Linezolid is an oxazolidinone antimicrobial drug active against antibiotic-resistant Gram-positive bacteria including methicillin-resistant *Staphylococcus aureus* (MRSA) and vancomycin-resistant enterococci [1]. Its high oral bioavailability provides a definite economic advantage compared with other MRSA therapeutic options, allowing earlier hospital discharge and reducing out-patient therapy cost. Linezolid is well-tolerated with nausea, vomiting, diarrhea and headache being the most commonly reported side effects [2]. Bone marrow suppression and neuropathies may occur in patients taking this antibiotic for more than two weeks.

Black hairy tongue (BHT) is a benign disorder characterized by hypertrophy and discoloration of the filiform papillae of the tongue [3]. This disorder has been associated with numerous medications and predisposing conditions. Linezolid-induced BHT has rarely been reported. This report examines a case of BHT believed to be caused by linezolid.

## Case presentation

A 56-year-old Caucasian man was admitted with acute-onset right-sided chest pain, shortness of breath, dry cough and fever. Chest X-ray findings were consistent with a right lower lobe pneumonia. Pulmonary computed tomography (CT) angiogram was negative for pulmonary embolism. After a three-day hospitalization, during which he received intravenous levofloxacin 750mg daily, he was discharged home on oral levofloxacin at 750mg daily. The patient was re-admitted four days later with worsening symptoms despite reporting compliance with antibiotic therapy. Follow-up chest imaging showed worsening right lower lobe infiltrate with new effusion. Failure to respond to levofloxacin raised concerns of multidrug-resistant bacteria. The patient was started on linezolid 600mg every 12 hours and meropenem one g every eight hours. He underwent a right-sided pleurocentesis to drain an uncomplicated exudative effusion. Blood and pleural fluid cultures were negative. The result of a human immunodeficiency virus serology test was negative. Urine legionella and histoplasma antigens were negative. After a six-day hospitalization, the patient’s condition improved and he was discharged home on intravenous ertapenem one g daily and oral linezolid 600mg every 12 hours to finish a 10-day course.

Eight days after discharge, the patient presented to the infectious disease clinic for follow up. He reported significant improvement in his respiratory symptoms and denied fever. He reported blackish discoloration of his tongue and dysgeusia. The patient was edentulous and denied brushing or regular mouthwash use. He denied drinking tea or the excessive consumption of coffee or other colored beverages. The patient was a cigarette smoker but had quit since his initial hospitalization 21 days prior. Aside from the above antibiotics, he was also taking hydrocodone and acetaminophen, five mg and 500mg, respectively, every six hours and albuterol and ipratropium bromide metered-dose inhaler every six hours.

**Figure 1:**
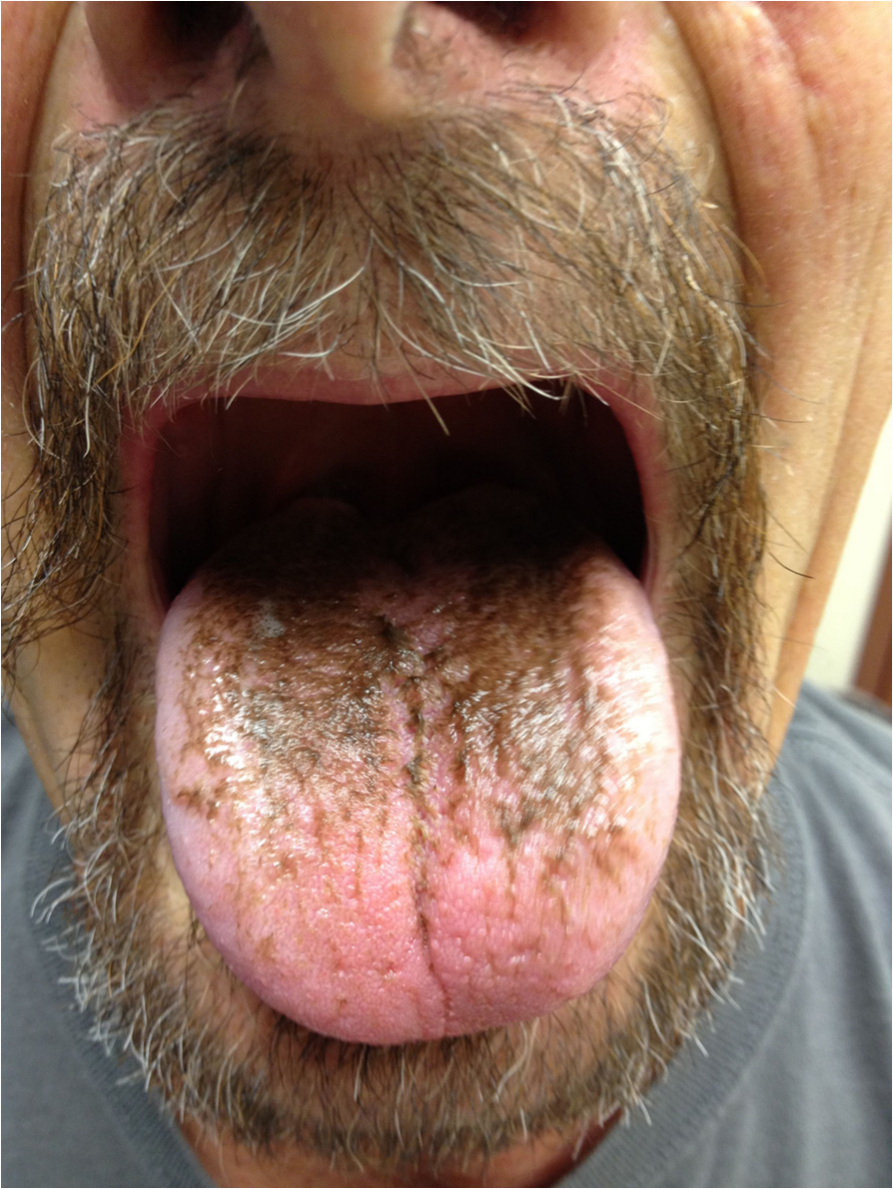
Brownish-black discoloration of the posterior two-thirds of the tongue after two weeks of linezolid therapy.

On examination, the patient was afebrile with normal heart rate and blood pressure. There was a brownish-black discoloration of his tongue that spared the tip and the sides (Figure 1). The discoloration could not be wiped by gauze. There was no discoloration of the buccal mucosa. There were no palpable cervical lymph nodes. He had no skin rash and his lung examination demonstrated significant improvement in air entry to the right lower lobe with minimal crackles.

**Figure 2:**
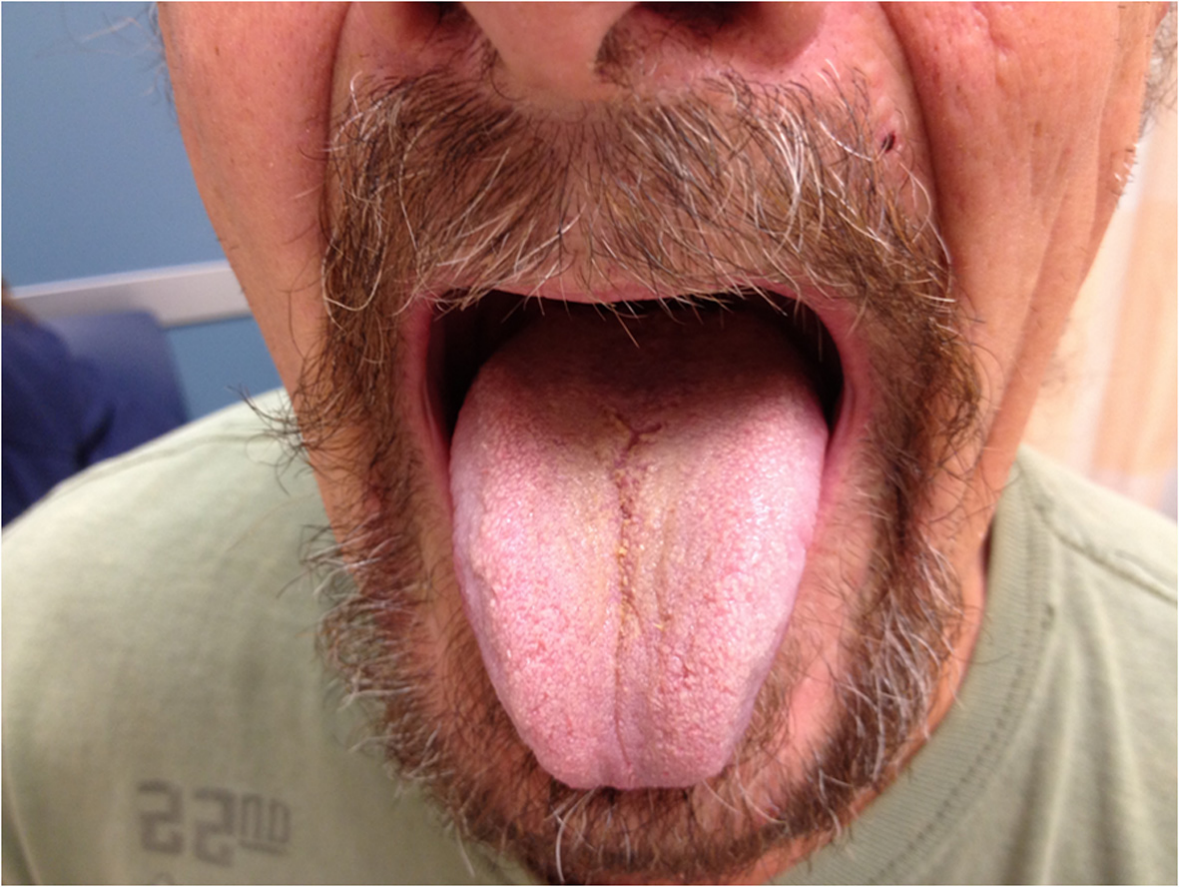
Complete resolution of the patient’s black hairy tongue four weeks after discontinuation of linezolid.

No blood tests were done. A swab for fungal and bacterial cultures grew normal oral flora. A liquid-based cytology preparation (ThinPrep smear) of the tongue showed food fibers but no yeast cells. The patient was diagnosed with BHT. He was reassured and was asked to finish his antibiotic course. He was directed to clean his tongue with a soft toothbrush and baking soda-containing toothpaste twice daily. A follow up visit four weeks later showed complete resolution of tongue discoloration (Figure 2).

## Discussion

Using the Naranjo Adverse Drug Reaction Probability Scale we present a case of probable linezolid-induced BHT in a 56-year-old man [4]. The patient’s concomitant medications have not been reported to cause BHT. Although a drug–drug interaction is a remote possibility, to the best of our knowledge, no such interactions have been reported. The occurrence of linezolid-induced BHT in our patient was probably aggravated by his poor oral hygiene. Other known causes were ruled out, including cigarette smoking because the patient had quit approximately three weeks prior to presentation.

Linezolid-induced BHT has been previously reported [5-9]. A MEDLINE search (from January 2000 to June 2012) revealed five reported cases. The median duration from initiating linezolid to the diagnosis of discoloration in the reported cases was two weeks (range: from two days to two weeks). The discoloration resolved after stopping linezolid in all reported cases after a median of seven days (range: from three days to six months). In two of the reported cases, the authors did not observe changes in the filiform papillae, labeling their patients with linezolid-induced tongue discoloration rather than BHT [5,6]. The possibility of early BHT, which might have been difficult to diagnose on clinical examination, was not entertained. The details of reported cases, including the current case, are presented in Table 1.

**Table 1:** Reported cases of linezolid-induced tongue discoloration and black hairy tongue

**Patient gender, age (years)**	**Clinical indication for linezolid therapy**	**Concomitantly used medications**	**Naranjo score**	**Ref #**
Male, 65*	Ampicillin-resistant enterococcal UTI in a kidney transplant recipient	Mycophenolate, tacrolimus, prednisone, TMP and SMX, nystatin, valgancyclovir	7	[5]
Male, 74	Staphylococcal skin infection in a lymphoma patient	NR	6	[7]
Female, 8**	MRSA septic arthritis	NR	6	[8]
Male, 42*	Empiric therapy for diskitis following laminectomy	Rifampin	6	[6]
Female, 40	Disseminated nocardial infection in a patient with SLE	Steroids, azathioprine, ceftriaxone, TMP and SMX	6	[9]
Male, 56	Empirical therapy for MRSA pneumonia	Ertapenem, hydrocodone and acetaminophen, albuterol and ipratropium bromide MDI	6	Current case

Abbreviations: MDI: metered-dose inhaler, MRSA: methicillin-resistant *Staphylococcus aureus*, NR: not reported, Ref #: reference number, SLE: systemic lupus erythematosus, TMP and SMX: trimethoprim and sulfamethoxazole, UTI: urinary tract infection.

*The authors did not observe filiform papillae changes on clinical examination and labeled their patients with linezolid-induced tongue discoloration not black hairy tongue.

**The authors described tongue discoloration but did not comment on the possibility of black hairy tongue.

The safety profile of linezolid has been well documented. In a study comparing linezolid and dicloxacillin for skin and soft tissue infections, the most frequently reported adverse events in the linezolid group were nausea (5.8%), headache (5.5%) and vomiting (3.3%) [2]. Longer courses of linezolid, more than two to four weeks, have been associated with reversible myelosuppression and neuropathy. In comparator-controlled trials for the treatment of skin and soft tissue infections, tongue discoloration associated with linezolid administration was reported in 1.1% of patients [9].

BHT is a self-limiting disorder characterized by abnormal hypertrophy and elongation of filiform papillae on the surface of the tongue [3]. There are no objective criteria for diagnosing this condition [10]. Although BHT is usually asymptomatic, occasionally patients may complain of tickling or burning of the tongue, nausea, halitosis, dysgeusia and unattractive appearance of the tongue [3]. The color black has been used to characterize this condition historically; however, brown, yellow and green discolorations have also been reported [3]. BHT is caused by defective desquamation of the dorsal surface of the tongue, usually in the posterior one-third [10]. This defective desquamation prevents normal debridement resulting in excessive growth and thickening of the filiform papillae that then collect debris, bacteria, fungi or other foreign materials which contribute to the discoloration. The exact mechanism of drug-induced BHT is unknown.

Several factors have been implicated in causing and/or aggravating BHT; these include smoking or chewing tobacco, drinking alcohol, poor oral hygiene, smoking crack cocaine or other street drugs, using peroxide-containing mouthwash, radiation therapy, trigeminal neuralgia, using drugs that cause xerostomia like anticholinergics, antihypertensives and antidepressants, and antibiotics such as tetracyclines and penicillins [11].

The diagnosis of BHT relies on the visual identification of discolored, elongated, and hypertrophied filiform papillae. Taking a detailed history is important in identifying contributing factors. Once BHT is diagnosed, discontinuation of the offending agent alongside practicing good oral hygiene usually resolves the problem [11]. Gentle tongue debridement can be accomplished by cleaning the tongue with a soft toothbrush and a solution of 3% hydrogen peroxide or baking soda [3].

## Conclusion

Linezolid-induced BHT is an uncommon, benign, self-limiting disorder. Other antibiotics, drugs that cause xerostomia, poor oral hygiene, smoking, and consumption of coffee or other colored beverages are known or probable risk factors. A thorough history should be obtained to rule out these risk factors in the setting of concomitant linezolid use. The prospective counseling of patients on the importance of good oral hygiene when taking this medication may be warranted. Further studies are needed to identify the exact mechanism of linezolid-induced BHT and to develop objective criteria for establishing the diagnosis.

## Consent

Written informed consent was obtained from the patient for the publication of this case report and accompanying images. A copy of the written consent is available for review by the Editor-in-Chief of this journal.

## Competing interests

The authors declare that they have no competing interests.

## Authors’ contributions

FAK collected patient data and prepared the first draft of the paper. DFM and JAZ reviewed the literature and edited the manuscript. All authors read and approved the final manuscript.
